# Mare’s and Cow’s Milk Fortified with Flaxseed Oil Through Freeze-Drying Microencapsulation: Physicochemical and Nutritional Properties

**DOI:** 10.3390/foods14020280

**Published:** 2025-01-16

**Authors:** Jolanta Gawałek, Dorota Cais-Sokolińska, Joanna Teichert

**Affiliations:** Department of Dairy and Process Engineering, Faculty of Food Science and Nutrition, Poznań University of Life Sciences, Wojska Polskiego 31/33, 60-624 Poznań, Poland; cais@up.poznan.pl (D.C.-S.); joanna.teichert@up.poznan.pl (J.T.)

**Keywords:** microencapsulation, milk, freeze drying, milk powders, flaxseed oil, functional food, bioactive foods

## Abstract

The microencapsulation via freeze drying of flaxseed oil in cow and mare milk was analyzed. The physicochemical and nutritional properties of the four obtained freeze-dried powder products were comparatively analyzed: microencapsulated and fortified with flaxseed oil cow milk (CMFO) and mare milk (MMFO), as well as pure cow milk (CM) and pure mare milk (MM). The moisture content, water activity, particle size distribution (PSD), loose and tapped bulk densities, flowability, color, and fatty acid profiles of the freeze-dried powders as well as the PSD of reconstituted emulsion droplets were investigated. For both types of milk, the fortified products achieved lower moisture content and water activity, higher loose and tapped bulk densities, better flowability, and lower particle sizes for the reconstituted emulsion droplets. The PSDs of the powders and reconstituted emulsion droplets showed significantly lower levels for the mare milk products than the cow milk products. The atherogenic index (AI) and thrombogenic index (TI) of cow milk products were three and six times higher than those for mare milk products, respectively. In both types of milk, fortified products did not show differences in their AI and TI values, except for the TI for cow milk (where CMFO < CM). The hypercholesterolemia fatty acid index (HcFA), hypocholesterolemic acids (DFAs), hypercholesterolemic acids content (OFA), and n-6/n-3 fatty acid ratio showed greater health benefits from mare milk products. Fortification with flaxseed oil showed increased health-promoting properties in the case of the DFA and OFA parameters (for mare milk) and n-6/n-3 ratio (for cow milk).

## 1. Introduction

Mare milk has been consumed by humans in various cultures for centuries, and the popularity of this milk is still growing, especially in some parts of Europe. It is worth noting that the composition of mare milk is significantly different from that of cows and other animals [1]. Mare milk is characterized by its protein content (from 15 to 28 g kg^−1^), a large quantity of lactose (from 58 to 70 g kg^−1^), and low levels of fat (from 3 to 20 g kg^−1^) [2]. Mare milk exhibits a distinct fatty acid profile, with a balanced distribution of saturated, monounsaturated, and polyunsaturated fatty acids. Therefore, it is worth emphasizing that mare milk has a higher percentage of unsaturated fatty acids (about 53% total fatty acids), which is similar to human milk (59.5%) and greater than that of cow milk (37%) [3].

The presence of conjugated linoleic acid (CLA) in mare milk has been of particular interest due to its potential health-promoting effects. While mare milk does provide various fatty acids, the overall nutritional content and potential health benefits should be considered in the context of an individual’s dietary needs. According to [3], mare milk, because of its structural and functional properties, can be used in human nutrition. Recent scientific findings on equine milk’s composition demonstrate its positive effects on patients with osteoporosis (by increasing calcium absorption), bronchial and lung diseases, stomach ulcers, cirrhosis, and anemia [3].

Fortifying mare milk with omega-3 fatty acids, such as alpha-linolenic acid (ALA), can enhance its nutritional value. The development of different diseases, including Alzheimer’s disease, cardiovascular disease, and inflammation, may be affected by the proportions of omega-3 and omega-6 fats in one’s diet [4]. For those who may have difficulty obtaining enough omega-3 fatty acids through their diets, supplementation plays a crucial role in maintaining health, and they are particularly important for older adults.

Flaxseed oil is extracted from the seeds of the flaxseed plant (*Linum usitatissimum* L.), most often by cold pressing to preserve its beneficial nutritional properties. Freshly pressed flaxseed oil is characterized by a yellowish color, a mild characteristic smell, and a nutty taste, which may become bitter during storage [5,6,7]. Flaxseed oil has been used for various applications since antiquity, but in the last three decades, flaxseed and its oil have been the focus of increased interest in the field of diet and disease research [8]. This is due to the chemical composition of flaxseed and flaxseed oil, rich in biologically active ingredients. Flaxseed is a rich source of polyunsaturated fatty acids, dietary fibers, proteins, lignans, phenolic acids, flavonoids, vitamins (A, C, F, and E), and minerals (P, Mg, K, Na, Fe, Cu, Mn, and Zn) [8,9,10]. The greatest pro-health advantage of flaxseed oil is the high content of omega-3 acids, particularly alpha-linoleic acid (ALA), which causes health-beneficial action in many diseases such as coronary heart disease, diabetes, and cancer [9,11]. In addition, the ALA content in flaxseed oil is beneficial for infant brain development, blood lipid reduction, arthritis, gastrointestinal disorders, eczemas, hypertension, and immune response disorders [9,11,12,13]. Due to these properties, the food industry uses flaxseed oil for food fortification, such as Indian yogurt [14], powder soup [15], ice cream [16], bread [17], and milk [18].

However, the disadvantage of flaxseed oil is its high sensitivity to oxidation (particularly its ALA component) [9,11], which significantly hinders effective food fortification while preserving the beneficial properties over a longer shelf life. Effective solutions to this problem are various types of microencapsulation processes which enable obtaining microcapsules of flax seed oil with greatly improved oxidative stability. The microcapsules obtained in this way are ideally suited for food fortification, particularly dried food.

Microencapsulation is a technological process commonly applied in the areas of food, pharmacy, and biotechnology, protecting active substances against external factors (heat, moisture, light, and oxygen), preventing their evaporation and volatilization, allowing masking an unpleasant taste or smell, preventing interaction with other compounds, or controlling the release of the core material [11,19,20]. The process consists of surrounding the active substance, usually called the core or inner phase, with a material or mixture of materials, usually called the outer phase, including the shell, carrier, matrix, encapsulating agent, or wall material [11,20,21]. The encapsulated core material can be in various forms—liquid, solid, or gaseous particles—while the encapsulating agent in solid form creates a tight barrier, completely isolating the core material from the external environment [20]. There are many methods of microencapsulation, from which three groups can be distinguished: chemical methods (e.g., interfacial polymerization), physical methods (e.g., spray drying, freeze drying, and fluid bed coating), and physicochemical methods (e.g., coacervation and ionotropic gelation) [9]. The most commonly used methods in the production of food ingredients are spray and freeze drying, in which water solutions, emulsions, or dispersions are dehydrated. Due to their specificity, both methods allow achieving a high degree of bioactivity retention of the core material (spray drying due to its short drying time and freeze drying due to the low material temperature and lack of air during drying (sublimation under vacuum conditions)). However, as reported in the literature, freeze drying achieves higher-quality microencapsules than spray drying, both in terms of encapsulation of flaxseed oil [11] and preservation of the bioactive and nutritional properties of milk [22,23,24]. Therefore, the present study focused on microencapsulation via freeze drying.

The research results published by Cais-Sokolińska et al. [25] unequivocally confirmed the possibility of freeze drying mare milk and then using the freeze-dried product to create good emulsions or foods with a low degree of aeration. Taking into account the above issues, the following hypothesis was formulated in this study: Microencapsulation of flaxseed oil in the mare milk matrix with the freeze-drying method contributes to improvement in the foaming, emulsifying, and health-promoting properties due to the change in the lipid profile.

In light of the above, the aim of this study is to evaluate the effectiveness and usefulness of fortification of mare milk with flaxseed oil as a result of microencapsulation through freeze drying and compare it with cow milk, a highly available milk. Both the obtained microcapsules of fortified milk and those reconstituted in an aqueous environment are evaluated. Useful and nutritional properties are analyzed in terms of obtaining milk drinks with specifically designed pro-health values.

## 2. Materials and Methods

### 2.1. Raw Milk

Mare milk was obtained from mares (Polish Coldblood) reared on an equine farm located in western Poland (Wielkopolska province). It was bulk milked from several mares milked on the same day to the amount of 10 L. The milk was collected in the fourth month of lactation, with foaling having occurred in February. By the fourth month of lactation, the milk’s chemical composition had already stabilized, and it was consistent in terms of density and acidity. The basic composition of the raw mare milk was as follows: fat content of 15.5 g/kg, total protein of 23.7 g/kg, lactose content of 63.1 g/kg. The milk was not centrifuged to change the fat content. The cow milk to the amount of 10 L was obtained from a farm from Holstein-Friesian cows, which were also from the same region of Wielkopolska. Both the mare milk and cow milk were collected during the same period (i.e., in June) at intervals of five days. A total of six milk collections were conducted during the month. The raw cow milk was centrifuged (Milky FJ 130 EPR separator, Janschitz GmbH, Althofen, Austria), and the fat content was normalized to 1.5% to reduce the content of this component, as in the mare milk. In the raw cow milk, after normalization of the fat content from 42.3 to 15.6 g/kg, the contents of the other basic components was as follows: total protein of 34.2 g/kg and a lactose content of 47.7 g/kg. Neither the mare milk nor the cow milk were homogenized; they were only cooled and kept for a maximum of 12 h after milking in separate SKM50 tanks from Plevnik (Dobrova, Slovenia). Both types of milk were pasteurized at 85 °C for 10 min in a Milky FJ 15 pasteurizer (Janschitz GmbH, Althofen, Austria). The experiment was performed 6 times (*n* = 6) (i.e., the total volume of mare milk used was 6 × 10 L, and that for the cow milk was 6 × 10 L). The authors’ assumption was to refer to earlier scientific achievements in the field of foaming and other useful qualities of lyophilized mare milk published in 2023 [25] by the same authors, and hence the raw materials and analytical methods used were the same.

### 2.2. Fortification of Raw Milk with Flaxseed Oil

In order to fortify the milk, 2% (*v*/*v*) flaxseed oil (Bunge, Kruszwica, Poland) was added to fresh cow or mare milk, pre-mixed, and homogenized at 15,000 rpm within 5 min using a TK-25 homogenizer (IKA Werke GmbH, Staufen, Germany). In total, 800 g of emulsions were prepared in 100 g portions. The emulsions prepared in this way were frozen immediately after homogenization of each portion.

### 2.3. Freeze Drying

Fresh cow and mare milk (CM and MM) and fortified cow and mare milk with flaxseed oil (CMFO and MMFO) were frozen using silicone cylindrical molds with dimensions of ϕ 25 × 10 mm to −40 °C (in an RLHE0845 freezer; Labcold Ltd., Chineham, UK) prior the freeze-drying process. Approximately 160 silicone cylindrical molds were used to freeze each milk product into discs. After being frozen for 24 h, the milk product discs were removed from the molds and arranged in a single layer on the trays of a freeze dryer. The freeze-drying procedure was performed on a semi-industrial scale using a CHWC-20A freeze dryer (Lyo-Tech Sp.z o.o., Międzyrzecz, Poland) to replicate an industrially relevant process. During the operation, the vacuum pressure was maintained consistently within the range of 60–70 Pa. The temperature of the heating plates was gradually adjusted, starting at 90 °C and decreasing to 40 °C by the end of the process. The entire freeze-drying process was completed within 16 h. Freeze-dried milk products, in the form of partially self-disintegrating fragile agglomerates of various sizes, were collected from the trays and subjected to a thorough powdering process. For this purpose, the products were crushed using an AGN grinder (Alexanderwerk AG, Remscheid, Germany) with a sieve (1.0 mm mesh size). The resulting powders were placed in sealed foil laminate pouches and kept at a temperature of 3 ± 0.5 °C for further analysis, with storage not exceeding two weeks. To ensure stability, the freeze-dried milk products were shielded from light during storage. However, measurements were conducted under brief exposure to light conditions (450 lx, f 590 cd, 120 lm) using a TES-1335 device (TES Electrical Electronic Corp., Taipei, Taiwan).

### 2.4. Samples for Physicochemical Testing

Four different milk product samples were subjected to physicochemical tests: cow milk (CM), mare milk (MM), cow milk fortified with flaxseed oil (CMFO), and mare milk fortified with flaxseed oil (MMFO). All milk product samples, depending on the physicochemical tests performed, were used in three different forms: raw, freeze-dried, and reconstituted freeze-dried samples. In the case of the reconstituted milk products, the freeze-dried powders were dissolved in distilled water at 18 °C in proportions to achieve the appropriate concentrations of raw milk products. Thus, 29.7, 24.2, 35.4, and 30.1 g of freeze-dried CM, MM, CMFO and MMFO, respectively, were dissolved in 250 mL of distilled water.

### 2.5. Moisture Content and Water Activity

The moisture contents of the freeze-dried milk products were measured with an HX204/M halogen apparatus (Mettler Toledo, Columbus, OH, USA) at 80 °C, while the determination of water activity was performed using an AquaLab Series 4TE device (Decagon Devices Inc., Pullman, WA, USA).

### 2.6. Particle Size Distribution (PSD) of Powder and Emulsion Droplets

The size distribution of the particles (PSD) was determined using a Mastersizer 2000 analyzer (Malvern Instruments Ltd., Malvern, UK), which is based on the laser diffraction method. Both measurements were carried out using the wet method with a Hydro 2000 G unit equipped with a high-speed stirrer and an ultrasonic source. In the PSD measurement of the powders, isopropanol was used as the dispersant, while the PSD measurement of the emulsion droplets was carried out in the prepared emulsion of reconstituted milk products, where water was used as the dispersant. After preparation of reconstituted freeze-dried milk product samples (methodology described in Section 3.4), each sample was homogenized at 15,000 rpm within 5 min using a TK-25 homogenizer (IKA Werke GmbH, Staufen, Germany). Three percentiles (D_10_, D_50_ and D_90_), the volume-weighted mean diameter D[4,3], and the span (Equation (1)) of the volume distribution were determined:

(1) $$span=\frac{D_{90}-D_{10}}{D_{50}}$$

### 2.7. Bulk Density and Flowability

The loose bulk density (ρ_l_) and tapped bulk density (ρ_t_) were measured according to ASTM D6393 [26] using a Thyr 2 vibrating laboratory apparatus (Saskia Hochvakuum und Labortechnik GmbH, Ilmenau, Germany) with appropriate adjustments. The flowability of the obtained freeze-dried powders was determined using the Hausner ratio (HR), which indicated the cohesiveness of the powders, and the Carr index (CI), which indicated the compressibility of the powders. The HR and CI were calculated according to Equations (2) and (3), respectively:

(2) $$HR=\frac{\rho_{t}}{\rho_{l}}$$

(3) $$CI=\frac{\rho_{t}-\rho_{l}}{\rho_{t}}\cdot 100\%$$

### 2.8. Color

The color values of the powders in the CIE L* a* b* system were measured with the reflectance method using an SP-60 spectrophotometer (X-Rite, Grandville, MI, USA) in the SPIN configuration. The L* value indicates the degree of lightness (scale: 0–100), a* indicates the red (positive values) and green (negative values) color measurements, and b* indicates the yellow (positive values) and blue (negative values) color measurements.

### 2.9. Fatty Acid Profile

The fatty acid profile was determined according to the methodology described by Cais-Sokolińska et al. [25]. The atherogenic index (AI), thrombogenic index (TI), hypercholesterolemia fatty acid index (HcFA), content of fatty acids with the hypocholesterolaemia effect (DFA), and hypercholesterolemia fatty acids (OFAs) of the fat milk samples were determined in accordance with the good laboratory practices and methodologies described in other publications [25,27,28].

### 2.10. Statistical Analysis

All determined physicochemical parameters were expressed as mean values ± standard deviation (SD) based on the measurements of 3 replicates at minimum. Statistical differences in the results for different milk products were verified by applying one-factor analysis of variance (ANOVA). Tukey’s HSD test was selected as the post hoc test, and the significance level was α = 0.05. Calculations were performed using Statistica software v. 13.1 (StatSoft Poland, Cracow, Poland).

## 3. Results and Discussion

### 3.1. Moisture Content (MC) and Water Activity (WA)

The values for the moisture content and water activity of the obtained freeze-dried cow and mare milk products are summarized in Table 1. All products had low values for the WA (0.21–0.37) and MC (1.30–1.62%). This shows that for all products, sufficient microbiological stability was achieved. The MC values of the freeze-dried cow milk (CM) and cow milk fortified with flaxseed oil (CMFO) were higher than their counterparts based on mare milk (MM and MMFO). In the case of WA, the same relationship was observed only for pure milks, whereas for the fortified milks, the differences were statistically insignificant. Moreover, in both kinds of milk, the process of microencapsulation of flaxseed oil resulted in achieving lower WA and MC values for the freeze-dried products than the freeze-dried raw milk counterparts. Other researchers obtained moisture results for various dairy products at similarly low levels, although slightly higher than ours (for example, 3.14–4.20% for cow milk [29], 2.44–3.0% for camel milk [30] and 3.1% for donkey milk [31]). On the other hand, the water activity in freeze-dried cow and camel milk was studied by Sulieman et al. [32], obtaining results quite similar to ours (0.148–0.326 and 0.253–0.334, respectively).

### 3.2. Particle Size Distribution (PSD) of Powders

**Figure 1:**
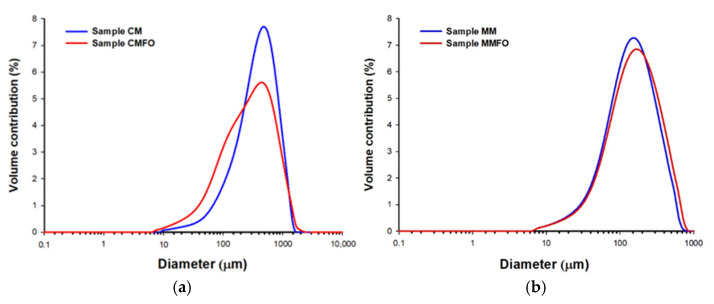
Graphical representation of particle size distribution of freeze-dried cow (**a**) and mare (**b**) milk products. CM = freeze-dried raw cow milk, CMFO = freeze-dried cow milk fortified with flaxseed oil, MM = freeze-dried raw mare milk, MMFO = freeze-dried mare milk fortified with flaxseed oil.

The particle size of food powders, including those achieved by freeze-drying, is a crucial attribute which influences a number of physicochemical properties of the powder (e.g., bulk density, flowability, compressibility, solubility, and hygroscopicity) [33]. Figure 1 shows a graphical representation of the cumulative particle size distribution (PSD) of the freeze-dried cow and mare milk products, while Table 2 summarizes the parameters characterizing this distribution. For all products, a unimodal PSD was obtained, where in the case of cow milk products (CM and CMFO), they were shifted further toward higher values compared with the distributions of mare milk products (MM and MMFO). This is also confirmed by the D_10_, D_50_, and D_90_ values presented in Table 2. The larger particle sizes for CM and CMFO indicate the formation of a more compact and durable solid structure during freeze drying than in the case of MM and MMFO products. This in turn results in easier crushing and disintegration into smaller particle sizes for mare milk products than cow milk using the same powdering method. These different behaviors of freeze-dried products are directly related to the differences in composition between cow and mare milk, particularly for the content of lactose and lipids [34].

Fortification of mare milk with flaxseed oil did not cause a significant change in the graphic image of the PSD (Figure 1b), in contrast to the cow milk products (Figure 1a), in which the PSD of CMFO was characterized by a larger spread than that of CM. This is also reflected in the increase in span value from 1.93 to 2.69 (Table 2). Moreover, the particle sizes of CMFO was smaller than that of CM (Table 2), which may indicate that the fortification of cow milk with flaxseed oil changed the structure of the solids to be less compact and more susceptible to mechanical disintegration.

In the case of determining the PSD with the volume-based laser diffraction method, the preferred parameter defining the mean particle diameter is the volume-weighted mean particle diameter D[4,3]. For the mare milk products, D[4,3] values were obtained which differed little from each other (i.e., 163.0 µm (MM) and 181.2 µm (MMFO)), while for the cow milk products, the obtained values were definitely higher, and additionally, the difference in values between MM and MMFO was highly significant (415.4 µm versus 351.4 µm, respectively).

To sum up, the particle size of the freeze-dried mare milk fortified with flaxseed oil (MMFO) was slightly higher compared with that of the pure mare milk (MM), whereas in the case of cow milk, on the contrary, significantly higher particle sizes were observed for the pure milk (CM) than the fortified milk (CMFO).

### 3.3. Bulk Density and Flowability

**Figure 2:**
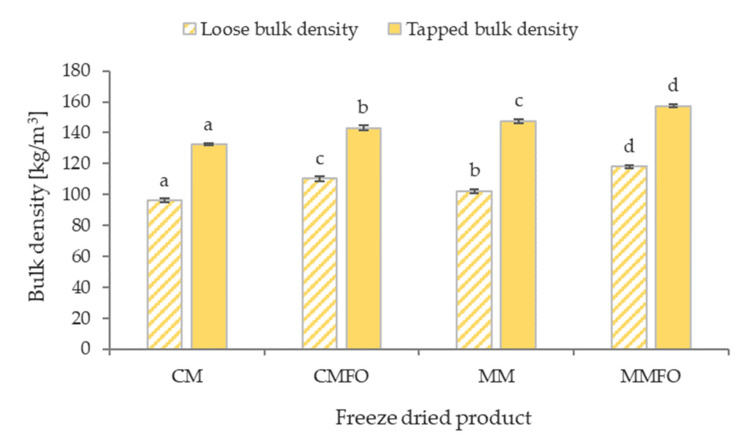
Loose and tapped bulk densities of freeze-dried cow and mare milk products. CM = freeze-dried raw cow milk, CMFO = freeze-dried cow milk fortified with flaxseed oil, MM = freeze-dried raw mare milk, MMFO = freeze-dried mare milk fortified with flaxseed oil. ^a, b, c, d^ Different letters show significant differences between mean values (*p* ≤ 0.05).

The bulk density and flowability of food powders are incredibly important utility properties from a technological point of view. The loose and tapped bulk density values are shown in Figure 2. The loose bulk density values were obtained in the range of 96–118 kg/m^3^, and the tapped bulk density values were between 132 and 157 kg/m^3^. Both mare milk products (MM and MMFO) achieved higher bulk densities than the corresponding cow milk products (CM and CMFO). It is also worth noting that higher values for the loose and tapped bulk densities were recorded for the products fortified with flaxseed oil (CMFO and MMFO) in comparison with the corresponding raw milk products (CM and MM). However, other researchers have achieved much higher values for both the loose and tapped bulk densities, such as levels of 310–370 kg/m^3^ and 380–450 kg/m^3^ for cow milk, respectively [32]. These differences may have resulted from different methods of grinding the freeze-dried milk (not given in the study by Sulieman et al. [32]) as well as from differences in the freezing rate, because in the compared case, shock freezing was used at an extremely low temperature (−75 °C). In turn, the high freezing speed resulted in the formation of fine ice crystals, which reduced the porosity of the freeze-dried products. This may consequently have led to higher bulk densities.

On the contrary, Baldelli et al. [35] achieved values for both bulk densities (loose and tapped) which were lower in the spray- and freeze-dried skimmed cow milk (37–39 kg/m^3^ and 62–81 kg/m^3^, respectively). In addition, the same study found that spray- and freeze-dried dairy products with higher fat contents achieved higher bulk densities (for example, 58 kg/m^3^ (loose) and 105 kg/m^3^ (tapped) for cream with 6% fat) [35]. These results confirm the trend observed in the present study after fortification of cow and mare milk with flaxseed oil, specifically that the bulk densities of CMFO and MMFO are higher than those of CM and MM (Figure 2).

Based on the bulk density values, Hausner ratios (HRs) and Carr indices (CIs) were determined, which are measures of the flowability and compressibility of powders, respectively (Table 3). Both the Hausner ratios and Carr indices for the mare milk products were higher than their cow milk-based counterparts. This indicates that freeze-dried products based on mare milk are characterized by higher compressibility and lower flowability than products based on cow milk. Moreover, it is worth noting that for both types of milk, a decrease in the HR and CI values was observed in the case of milk fortified with flaxseed oil via microencapsulation. In other words, this means that the improvement in powder flowability was a result of the microencapsulation process, which was indicated well by the use of Carr’s [36] classification of powder flowability. According to this classification, the pure milk products (CM and MM) were classified as having “poor” flowability, and the milk products fortified with flaxseed oil were classified as having “passable” flowability (Table 3). Sulieman et al. [32] obtained freeze-dried cow milk powder with significantly better flowability (“fair” and “good” (HR = 1.13–1.28)), which was related to a simultaneous higher loose bulk density. The potential causes of differences in the obtained powder flow properties were the same as in the case of differences in bulk density discussed above. Another example study [35] among scientific reports obtained spray- and freeze-dried powders of skimmed cow milk with much worse flowability (HR = 2.0, CI = 51%, or “very, very poor flowability” according to Carr’s classification [36]) in comparison with our study. This correlates with the bulk density values obtained by Baldelli et al. [35] described above. The same study [32] also conducted tests for cow milk products with different fat contents and showed a slight improvement in the flowability of spray- and freeze-dried powders in the fat content range of 2–10%. This is in line with the observed trend in this study in the case of increasing the fat content in products fortified with flaxseed oil.

### 3.4. Color

The CIE Lab system color scale of the freeze-dried cow and mare milk products was analyzed. The results are presented in Table 4. The freeze-dried cow milk products were characterized by a higher L* brightness compared with the mare milk products, and in both cases, fortification of pure milk with flaxseed oil did not result in a statistically significant change in the L* color brightness. The values of the a* color parameter, defining the balance between green and red for all of the tested products, were at a similar level, except for freeze-dried fortified cow milk (CMFO), for which the a* value was slightly higher. In addition, the cow milk products were characterized by a color shifted more toward yellow than mare milk products (higher values for the b* parameter).

To sum up, fortification of the cow milk with flaxseed oil slightly changed the color of the freeze-dried powder toward red and yellow, whereas fortification of the mare milk only rather slightly changed its color toward yellow.

The determined color parameters for the freeze-dried cow and mare milk were compared with the results of other studies. Milovanovic et al. [37] determined the mean values of the color parameters for both milks based on scientific reports from across 10 years. For raw cow milk, the parameters L* = 81.0 ± 8.1, a* = −1.5 ± 3.0, and b* = 7.5 ± 4.4 were obtained, and for raw mare milk, L* = 73.5 ± 14.3, a* = −2.2 ± 0.2, and b* = −2.3 ± 2.4 were obtained. In relation to these values, the obtained color parameters of the freeze-dried cow milk in this study were similar, taking into account the confidence intervals, while for the freeze-dried mare milk, only the lightness L* was similar. On the other hand, the parameters a* and b* symbolize the shift of the color toward red and yellow. In summary, the differences in color between raw milk and freeze-dried powder were insignificant in the case of cow milk, while in the case of mare milk, the color change was distinct, most likely due to the higher content of lactose, which is sensitive to Maillard reactions and may cause color changes.

Moreover, Milovanovic et al. [37] also determined the mean values of the color parameters for cow milk powder products based on 17 worldwide scientific reports, obtaining L* = 88.6 ± 10.1, a* = 0.2 ± 6.8, and b* = 11.8 ± 6.1. A comparison of these values with the color parameters of the obtained freeze-dried cow milk also showed similarity, with a particularly higher degree of similarity for the yellowness b*.

### 3.5. Particle Size Distribution (PSD) of Emulsion Droplets

**Figure 3:**
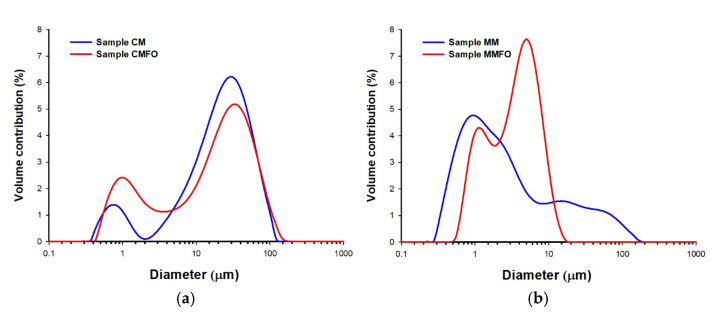
Graphical representation of the particle size distribution of emulsion droplets of reconstituted freeze-dried cow (**a**) and mare (**b**) milk products. CM = freeze-dried raw cow milk, CMFO = freeze-dried cow milk fortified with flaxseed oil, MM = freeze-dried raw mare milk, MMFO = freeze-dried mare milk fortified with flaxseed oil.

Both reconstituted cow milk products were characterized by significantly larger emulsion droplet sizes, as indicated by both the PSD graphic images (Figure 3) and the values of the mean droplet diameter D[4,3] (Table 5). For example, in the case of fortified products, the droplet size of the cow milk emulsion (CMFO) was more than six times larger than that of the fortified mare milk emulsion (MMFO). The differences in the emulsion droplet sizes may be related to differences in the chemical compositions of cow and mare milk, particularly the differences in lactose and protein content. The higher protein content in cow milk products, especially casein, may require a longer rehydration time, and it should be emphasized that proteins are effective emulsion stabilizers in raw milk. According to the adopted methodology for reconstituting freeze-dried dairy products, the rehydration and homogenization time was short (10 min), and hence the cow milk products with a high content of difficult-to-hydrate proteins (especially casein) did not show a state of high emulsification with small droplet sizes, in contrast to the mare milk products. In this case, a lower protein content, as well as another type of protein, probably rehydrates faster and supports the formation of a subtle emulsion with rather small emulsion droplet sizes.

### 3.6. Fatty Acid Profile

The atherogenic index (AI) of the freeze-dried cow milk (CM) and freeze-dried cow milk fortified with flaxseed oil (CMFO) was three times higher than in the case of using mare milk (Table 6). However, it was shown that the microencapsulation process associated with the addition of flaxseed oil did not significantly improve the health-promoting properties of the fat related to the atherogenicity of either type of milk. This shows that the addition of flaxseed oil was too small to change the effect of saturated fatty acids. It is specifically the saturated fatty acids C12:0, C14:0, and C16:0 which contribute to the increase in the AI (i.e., they have an atherogenic effect and thus cause an increase in the concentration of total cholesterol and the LDL fraction). The AI of cow milk varies depending on the breed, but it is usually in the range of 2.08–5.13 [2]. The proportions of C14:0, C16:0, and C18:0 in the fat were higher in the cow milk than in the mare milk, which resulted in 5–6 times higher thrombogenic index (TI) values (*p* < 0.05). However, fortification of the cow milk with flaxseed oil reduced the TI value by approximately 17% (*p* < 0.05). The TI is an indicator of stimulation of platelet activity and aggregation. The n-6 PUFAs have a strong antiatherogenic effect by reducing blood plasma lipids, while n-3 PUFAs have an antithrombogenic effect by reducing platelet activity. Hence, the analysis of the health-promoting potential of the fat of innovative products should take into account the ratio of n-6 to n-3 acids. Fatty acids are considered to be regulators of biological processes in various tissues and affect the functions of cell membranes. In general, the composition of milk fat determines not only its health-promoting value but also its sensory characteristics and ability to be further processed [38].

The assessment of the share of identified fatty acids with hypercholesterolemic activity (HcFa) increasing the cholesterol level clearly indicates that mare milk is considered to be more health-promoting (Table 7). The share of these acids compared with cow milk (CM and CMFO) was on average 1.7 times lower (*p* < 0.05). When analyzing the biological potential of mare milk fat, it was found to contain higher amounts of hypocholesterolemic acids, namely DFAs, which reduce cholesterol levels (DFA = 53.64 in MM), compared with cow milk (39.79 in CM). They are considered highly desirable in the human diet. The process of microencapsulation of mare milk also increased the health-promoting value (ΔDFA = 2.33, *p* < 0.05). This tendency was not demonstrated as a result of the microencapsulation of cow milk (*p* > 0.05).

Based on the analysis of the content of n-6 and n-3 acids in the fat, more equal proportions were found in the case of mare milk. The samples of freeze-dried mare milk and freeze-dried mare milk fortified with flaxseed oil did not differ in terms of the n-6/n-3 ratio. However, it was shown that microencapsulation could reduce the n-6/n-3 ratio, but only in the case of cow milk (*p* < 0.05). According to the recommendations for proper nutrition, the proportion of acids from the n-6 to n-3 family in the diet should be (4–5):1. The n-6 and n-3 FAs are precursors for eicosanoids such as prostaglandins, leukotrienes, and resolvins. These immune molecules have strong proinflammatory, weak proinflammatory, or anti-inflammatory potential [39]. The enzymes involved in the conversion of n-6 and n-3 fatty acids into their respective precursors, as well as in the production of eicosanoids, are identical regardless of the fatty acid type. For this reason, a high ratio of n-6 to n-3 may cause a shift toward the synthesis of pro-inflammatory eicosanoids, which results in a stronger inflammatory response. Hence, too much disproportion between these families of acids may disturb the balance in the amount of synthesized eicosanoids, often acting antagonistically. Such a state further leads to various pathological conditions which disrupt the proper functioning of the body. Hence, it is advisable to use diets with a lower n-6/n-3 ratio [40]. In addition, the ratio of n-6 to n-3 fatty acids not only affects maintenance of the inflammatory balance but also the maintenance of lipid metabolism and reductions in oxidative stress [41].

## 4. Conclusions

This study showed that freeze drying allows obtaining useful pure milk powder products as well as those enriched with oil.

The freeze-dried powders made from mare milk (both raw and fortified with flaxseed oil) showed better health-promoting properties in the nutritional areas of the fatty acid profile than the cow milk products. This was evidenced by the values of several indices, including the AI, TI, HcFA, DFA, and OFA, as well as the n-3/n-6 ratio.

For both types of milk, the products fortified with flaxseed oil had a lower moisture content, higher loose and tapped bulk densities, better flowability, as well as lower particle sizes for the reconstituted emulsion droplets than their pure counterparts. Additionally, fortification with flaxseed oil enhanced the health-promoting properties in the case of the DFA and OFA parameters (for mare milk) and the n-6/n-3 ratio (for cow milk).

The tested composite product, which was flaxseed oil microencapsulated via freeze drying in a mare milk matrix, showed good physicochemical, handling, and nutritional properties, which indicates its potential for use as a valuable, health-promoting food ingredient. However, one limitation may be the low availability of mare milk as well as the need to further determine the product’s stability regarding lipid oxidation.

## Figures and Tables

**Table 1:** Moisture content and water activity of freeze-dried cow and mare milk products.

Freeze-Dried Product	Moisture Content (wb, %)	Water Activity
CM	1.62 ± 0.03 ^c^	0.37 ± 0.01 ^c^
CMFO	1.45 ± 0.02 ^b^	0.20 ± 0.00 ^a^
MM	1.38 ± 0.03 ^ab^	0.30 ± 0.04 ^b^
MMFO	1.30 ± 0.01 ^a^	0.21 ± 0.00 ^a^

CM = freeze-dried raw cow milk, CMFO = freeze-dried cow milk fortified with flaxseed oil, MM = freeze-dried raw mare milk, MMFO = freeze-dried mare milk fortified with flaxseed oil, wb = wet basis. ^a, b, c^ Different letters show significant differences between mean values (*p* ≤ 0.05).

**Table 2:** Particle size distribution of freeze-dried cow and mare milk products.

Freeze-Dried Product	D_10_ (μm)	D_50_ (μm)	D_90_ (μm)	D[4,3] (μm)	Span
CM	104.4 ± 1.3 ^c^	363.5 ± 10.4 ^c^	805.0 ± 36.8 ^b^	415.4 ± 15.2 ^c^	1.93 ± 0.04 ^a^
CMFO	59.3 ± 0.8 ^b^	265.4 ± 9.7 ^b^	772.7 ± 22.1 ^b^	351.4 ± 10.9 ^b^	2.69 ± 0.06 ^c^
MM	45.0 ± 0.3 ^a^	133.4 ± 1.3 ^a^	326.9 ± 7.7 ^a^	163.0 ± 2.9 ^a^	2.11 ± 0.03 ^b^
MMFO	47.1 ± 0.4 ^a^	145.9 ± 2.8 ^a^	370.8 ± 11.7 ^a^	181.2 ± 4.6 ^a^	2.22 ± 0.03 ^b^

CM = freeze-dried raw cow milk, CMFO = freeze-dried cow milk fortified with flaxseed oil, MM = freeze-dried raw mare milk, MMFO = freeze-dried mare milk fortified with flaxseed oil. ^a, b, c^ Different letters show significant differences between mean values (*p* ≤ 0.05).

**Table 3:** Characteristics of freeze-dried cow and mare milk powders’ flowability: Hausner ratio (HR) and Carr’s index (CI).

Freeze-Dried Product	Hausner Ratio		Carr Index	
	(-)	Flowability *	(%)	Flowability *
CM	1.38 ± 0.02 ^c^	poor	27.4 ± 1.0 ^c^	poor
CMFO	1.30 ± 0.00 ^a^	passable	22.9 ± 0.2 ^a^	passable
MM	1.44 ± 0.01 ^d^	poor	30.6 ± 0.3 ^d^	poor
MMFO	1.33 ± 0.01 ^b^	passable	25.0 ± 0.6 ^b^	passable

CM = freeze-dried raw cow milk, CMFO = freeze-dried cow milk fortified with flaxseed oil, MM = freeze-dried raw mare milk, MMFO = freeze-dried mare milk fortified with flaxseed oil. ^a, b, c, d^ Different letters show significant differences between mean values (*p* ≤ 0.05). * Flowability of powders according to Carr’s [36] classification.

**Table 4:** Results for color parameters of freeze-dried cow and mare milk products.

Freeze-Dried Product	L*	a*	b*
CM	83.0 ± 0.4 ^b^	−0.84 ± 0.01 ^b^	11.9 ± 0.2 ^c^
CMFO	84.2 ± 0.4 ^b^	−0.61 ± 0.07 ^a^	14.0 ± 0.4 ^d^
MM	71.7 ± 0.7 ^a^	−0.82 ± 0.05 ^b^	8.1 ± 0.1 ^a^
MMFO	73.1 ± 1.2 ^a^	−0.80 ± 0.02 ^b^	9.2 ± 0.2 ^b^

CM = freeze-dried raw cow milk, CMFO = freeze-dried cow milk fortified with flaxseed oil, MM = freeze-dried raw mare milk, MMFO = freeze-dried mare milk fortified with flaxseed oil. ^a, b, c, d^ Different letters show significant differences between mean values (*p* ≤ 0.05).

**Table 5:** Particle size distribution of emulsion droplets of reconstituted freeze-dried cow and mare milk products.

Freeze-Dried Product	D_10_ (μm)	D_50_ (μm)	D_90_ (μm)	D[4,3] (μm)	Span
CM	2.1 ± 0.0 ^c^	20.8 ± 0.4 ^d^	55.6 ± 0.9 ^c^	25.7 ± 0.5 ^d^	2.57 ± 0.02 ^a^
CMFO	0.9 ± 0.0 ^b^	18.1 ± 0.4 ^c^	58.4 ± 0.9 ^c^	24.4 ± 0.5 ^c^	3.18 ± 0.02 ^a^
MM	0.5 ± 0.0 ^a^	1.8 ± 0.1 ^a^	30.7 ± 1.5 ^b^	9.9 ± 0.1 ^b^	16.25 ± 1.70 ^b^
MMFO	0.9 ± 0.0 ^b^	3.4 ± 0.1 ^b^	7.5 ± 0.1 ^a^	3.8 ± 0.1 ^a^	1.95 ± 0.02 ^a^

CM = freeze-dried raw cow milk, CMFO = freeze-dried cow milk fortified with flaxseed oil, MM = freeze-dried raw mare milk, MMFO = freeze-dried mare milk fortified with flaxseed oil. ^a, b, c, d^ Different letters show significant differences between mean values (*p* ≤ 0.05).

**Table 6:** Atherogenic and thrombogenic indices of freeze-dried cow and mare milk fat.

Freeze-Dried Product	AI	TI
CM	3.02 ± 0.11 ^b^	3.14 ± 0.08 ^c^
CMFO	3.00 ± 0.11 ^b^	2.62 ± 0.15 ^b^
MM	1.01 ± 0.03 ^a^	0.51 ± 0.07 ^a^
MMFO	0.98 ± 0.04 ^a^	0.49 ± 0.05 ^a^

CM = freeze-dried raw cow milk, CMFO = freeze-dried cow milk fortified with flaxseed oil, MM = freeze-dried raw mare milk, MMFO = freeze-dried mare milk fortified with flaxseed oil. ^a, b, c^ Different letters show significant differences between mean values (*p* ≤ 0.05). AI *=* atherogenic index (AI = (C12:0 + 4 × C14:0 + C16:0)/UFA), TI *=* thrombogenic index (TI = (C14:0 + C16:0 + C18:0)/[(0.5 × MUFA) + (3 × n-3) + (0.5 × n-6) + (n-3/n-6)]).

**Table 7:** Health-promoting properties of freeze-dried cow and mare milk fat.

Freeze-Dried Product	HcFA	DFA	OFA	DFA/OFA	n-6/n-3
CM	44.62 ± 1.41 ^b^	39.79 ± 1.48 ^a^	59.86 ± 1.41 ^c^	0.67 ± 0.04 ^a^	2.73 ± 0.22 ^c^
CMFO	42.34 ± 3.29 ^b^	40.09 ± 1.82 ^a^	59.91 ± 1.82 ^c^	0.67 ± 0.05 ^a^	1.67 ± 0.36 ^b^
MM	25.01 ± 0.68 ^a^	53.64 ± 0.91 ^b^	45.63 ± 0.92 ^b^	1.18 ± 0.04 ^b^	0.95 ± 0.14 ^a^
MMFO	25.01 ± 0.75 ^a^	55.97 ± 0.92 ^c^	44.04 ± 0.92 ^a^	1.27 ± 0.05 ^c^	0.97 ± 0.12 ^a^

CM = freeze-dried raw cow milk, CMFO = freeze-dried cow milk fortified with flaxseed oil, MM = freeze-dried raw mare milk, MMFO = freeze-dried mare milk fortified with flaxseed oil. ^a, b, c^ Different letters show significant differences between mean values (*p* ≤ 0.05). HcFA = hypercholesterolemic fatty acid index (HcFA = C14:0 + C16:0), DFA = hypocholesterolemic fatty acid content (DFA = Σ UFA + C18:0), OFA = hypercholesterolemic fatty acid content (OFA = Σ SFA—C18:0, where Σ UFA is sum of MUFA (C10:1, C12:1, C14:1 cis-9, C16:1 cis-9, C16:1 trans-9, C17:1 cis-9, C18:1 cis-9, C18:1 cis-11, C18:1 cis-12, C18:1 trans-9, C18:1 trans-11, and C20:1 cis-11) and PUFA (C18:2 cis-9, trans-11, C18:2 cis-9, cis-12, C18:3 cis-6, cis-9, cis-12, C18:3 cis-9, cis-12, cis-15, C20: 2n-6, C20: 3n-6, C20: 4n-6, C20: 5n-3, and C22: 5n-3) and Σ SFA is sum of C4:0, C6:0; C8:0, C10:0, C11:0, C12:0, C13:0, C14:0, C14:0 iso, C15:0, C15:0 iso, C15:0 anteiso, C16:0, C16:0 iso, C17:0, C17:0 iso, C17:0 anteiso, C18:0, C18:0 iso, C20:0, C22:0, and C24:0).

## Data Availability

The original contributions presented in the study are included in the article, further inquiries can be directed to the corresponding author.
